# Clinicopathologic features of invasive metaplastic and micropapillary breast carcinoma: comparison with invasive ductal carcinoma of breast

**DOI:** 10.1186/s13104-018-3623-z

**Published:** 2018-07-31

**Authors:** Atif Ali Hashmi, Saher Aijaz, Raeesa Mahboob, Saadia Mehmood Khan, Muhammad Irfan, Narisa Iftikhar, Mariam Nisar, Maham Siddiqui, Muhammad Muzzammil Edhi, Naveen Faridi, Amir Khan

**Affiliations:** 1 Liaquat National Hospital and Medical College, Karachi, Pakistan; 2 Shaheed Zulfiqar Ali Bhutto Institute of Science and Technology, Karachi, Pakistan; 3 Department of Statistics, Liaquat National Hospital and Medical College, Karachi, Pakistan; 4 Aga Khan University, Karachi, Pakistan; 5 Department of Pathology, National Hospital and Medical College, Karachi, Pakistan; 6 Department of Surgery, Brown University, Providence, RI USA; 7 Department of Medicine, Kandahar University, Kandahar, Afghanistan

**Keywords:** Metaplastic carcinoma, Micropapillary carcinoma, ER, PR, Her2neu, Breast cancer

## Abstract

**Objectives:**

The aim of this study was to determine the frequency of metaplastic breast carcinoma and invasive micropapillary carcinoma in our population and also to compare the clinico-pathologic features of metaplastic breast carcinoma and invasive micropapillary carcinoma with invasive ductal carcinoma, not otherwise specified (IDC, NOS).

**Results:**

86.9% of the cases were identified as ductal carcinoma, NOS, while 2.2% were metaplastic and 0.76% cases were micropapillary carcinoma. Metaplastic carcinomas were found to be of higher grade as compared to IDC, NOS as 81% of metaplastic carcinoma were grade III compared to 35% IDC, NOS. 79% of metaplastic carcinoma were ER negative and 86% were PR negative, respectively as compared to ductal carcinoma NOS, which were 40% ER negative and 54% were PR. Similarly, 86.7% micropapillary cancers were ER positive and 73.3% were PR positive. Moreover, 66.7% micropapillary carcinoma showed nodal metastasis and 77.8% showed lymphovascular invasion, which was significantly higher than that of IDC, NOS micropapillary and metaplastic carcinomas accounts for less than 2 and 1% of the breast cancer burden in our population and highly correlates with poor prognosis parameters therefore, require more intensive management in our population.

## Introduction

Due to heterogeneous nature of the breast cancer, understanding of potentially aggressive cancers helps in designing tailored therapeutic interventions as it assists in comprehending the prognosis of the disease. Metaplastic breast carcinomas (MBC) and invasive micropapillary breast carcinomas (IMBC) are two aggressive phenotypic variants of breast cancers with a low prevalence rate amongst the general population. Although these variants are relatively rare but pose significant challenge in terms of breast cancer treatment. Majority of the special subtypes of breast carcinoma including mucinous, tubular, cribriform etc. are more indolent in behavior than conventional ductal carcinoma, not otherwise specified, and confers relatively better prognosis and favorable outcomes [1–3]. Invasive micropapillary carcinoma is recognized by tubulo-alveolar formation, which is visible in empty spaces with fragile pseudopapillary structures devoid of fibro-vascular core scattered across the membrane [4]. This micropapillary differentiation is visible as tight clusters of tumor cells present in cleft like retraction spaces resembling lymphatics [5]. Clinical significance of micropapillary differentiation relies on high incidence of nodal metastasis [6]. Metaplastic carcinomas are a diverse group of tumors showing divergent differentiation towards squamous, spindle cell or mesenchymal. These tumors are found to be negative for hormone receptors and Her2neu and therefore exhibit a basal like breast cancer profile [7, 8]. As a consequence of systemic metastasis, they are regarded as one of the most aggressive breast tumors [9]. Epithelial to mesenchymal transition in these subtypes of breast carcinomas are proposed to be associated with the tendency of the tumor cells to acquire properties of stem cells [10]. Since these subtypes of breast cancers are less responsive to conventional hormonal and chemotherapeutic interventions, we aimed to evaluate the frequency of metaplastic breast carcinoma and invasive micropapillary carcinoma in our population with regard to clinico-pathologic features of invasive ductal carcinoma, not otherwise specified. Moreover, in the era of personalized therapy, knowledge of histologic and prognostic parameters of individual types of cancers is extremely important as these features may differ in different populations. Therefore, evaluating prognostic features of micropapillary and metaplastic breast cancer in this study will be helpful in individualizing treatment strategies in locoregional population.

## Main text

### Methods

**Fig. 1:**
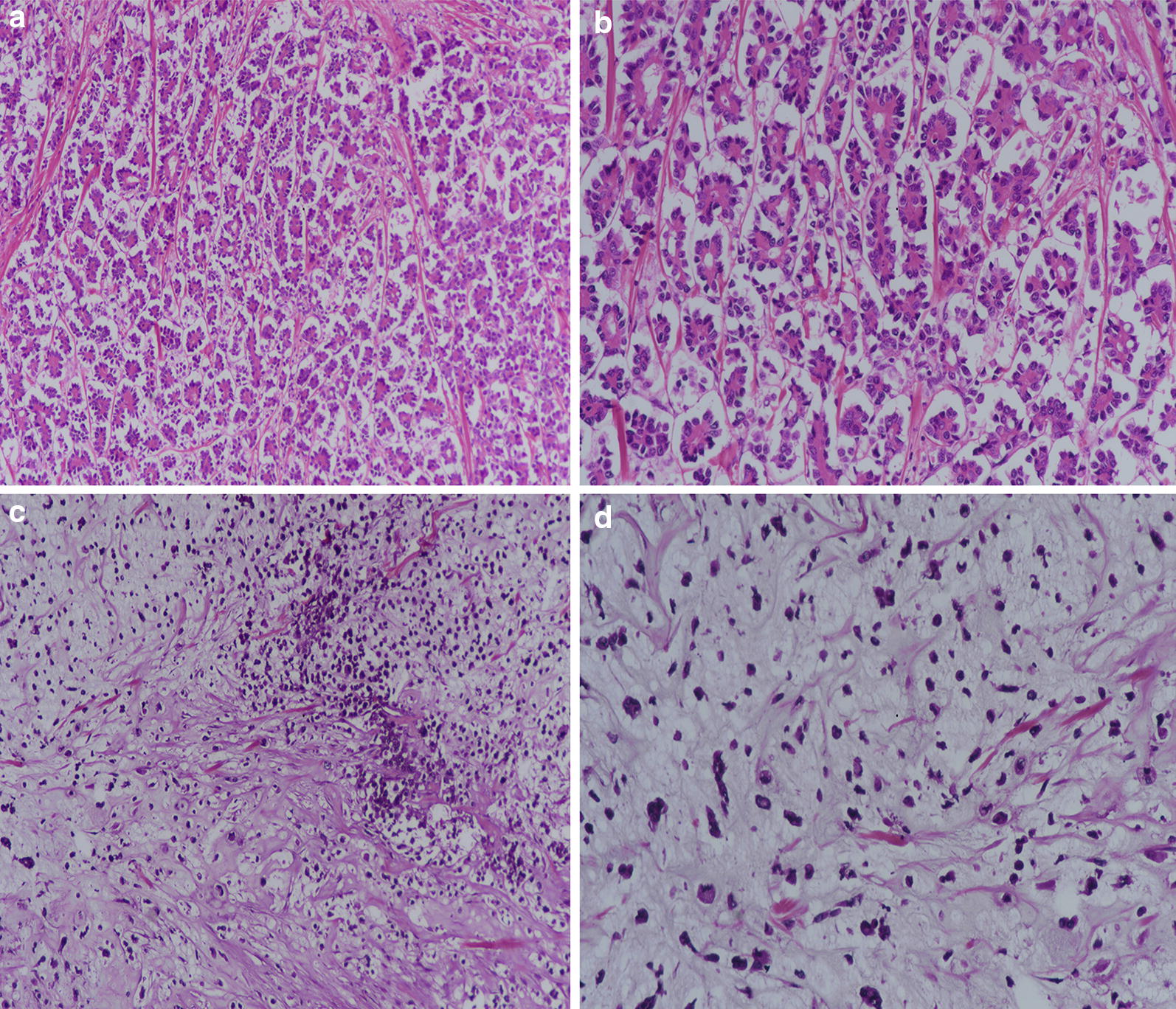
**a** Microscopic sections of Micropapillary carcinoma, ×40 showing tumor cells in cleft like retraction spaces. **b** Microscopic sections of Micropapillary carcinoma, ×200 showing reverse polarization of tumor cells. **c** Microscopic sections of Metaplastic carcinoma showing spindled tumor cells with myxoid matrix producing stroma, ×40 magnification. **d** Microscopic sections of Metaplastic carcinoma, ×200 magnification, showing tumor cells floating in chondromyxoid stroma

We reviewed 1951 cases of breast carcinoma treated at Liaquat National hospital from January 2008 till December 2014. The specimens include trucut biopsies, breast conservation surgeries and modified radical mastectomies. All the slides of these cases were retrieved and reviewed by two experienced pathologists. Micropapillary differentiation was defined as tumor nests present in cleft like spaces resembling lymphatic spaces. Micropapillary carcinomas were defined as tumors with > 90% micropapillary differentiation (Fig. 1). Metaplastic carcinomas were categorized as tumors showing spindle cell, squamous or mesenchymal differentiation (osseous, chondroid or matrix producing stroma) as shown in Fig. 1. Histopathologic characteristics recorded include tumor type, size, grade and lymph node status along with degree of necrosis, lymphocytic reaction and fibrosis. One representative section is selected for IHC studies including ER, PR, her2neu and ki67 by DAKO envision method. Following antibodies are used.

1. FLEX Monoclonal Rabbit Anti-human Estrogen receptor alpha, clone EP1, ready to use, purchased from DAKO (code IR084).
2. FLEX Monoclonal Mouse Anti-human Progesterone receptor, clone PgR 636, ready to use, purchased from DAKO (code IR068).
3. Polyclonal Rabbit Anti-human c-erbB-2 oncoprotein, purchased from DAKO (code A0485).
4. FLEX Monoclonal Mouse Anti-human, Ki67 Antigen, Clone MIB-1, Ready to use, purchased from DAKO (code IR626).

Immunohistochemistry was performed using DAKO envision kit according to manufacturers recommendations. Normal breast tissue was used as positive controls for ER, PR and as negative control for Her2neu. Known case of her2neu amplified breast cancer was used as positive control for Her2neu. For ki67 benign reactive lymph node was used as positive control.

More than 1% nuclear staining for ER/PR was considered positive expression.

HER2neu were scored based on the intensity and percentage of positive cells on a scale of 0 to 3 + according to ASCO/CAP guidelines [11, 12]. Cases with intermediate (2+) expression of Her2neu underwent subsequent FISH testing for Her2neu gene amplification. FISH testing was performed using FDA approved Path Vysion Her2 DNA Probe kit according and reported according to CAP guidelines.

Ki-67 immunoreactivity was recorded as continuous variables, based on the proportion of positive tumor cells (0–100%). At least 1000 cells were evaluated before calculating an average estimate. If hot spots were present, then they are included in the estimation. Ki67 index was further categorized into four groups, < 14%, 15–24%, 25–44%, > 45%.

Statistical package for social sciences (SPSS 21) was used for data compilation and analysis. Mean and standard deviation were calculated for quantitative variables. Frequency and percentage were calculated of qualitative variables. Chi square was applied to see association. *T* test was applied to compare difference in means among groups by considering P-value ≤ 0.05 as significant.

### Results

The study involved 1951 cases of primary breast carcinoma, out of which 1695 (86.9%) cases were ductal carcinoma, NOS, while 43 (2.2%) and 15 (0.76%) cases were of metaplastic and micropapillary carcinoma, respectively and thus included in the study. Table 1 shows the comparison of metaplastic carcinoma with ductal carcinoma, NOS.

**Table 1:** Association of clinico-pathologic parameters of metaplastic breast cancer subtype with Ductal carcinoma, NOS

	n (%)			P-value
	Metaplastic	Ductal, NOS	Total	
Age (years)^ab^	49.21 ± 12.92	50.54 ± 12.48	–	0.49
Age group (n = 1738)				
≤ 30	3 (3.3)	89 (5.3)	92 (5.3)	0.767
31–50	21 (48.8)	822 (48.8)	843 (48.5)	
51–70	16 (37.2)	692 (40.8)	708 (40.7)	
> 70	3 (7)	92 (5.4)	95 (5.5)	
Tumor grade (n = 1738)				
Grade 1	3 (7)	160 (9.4)	163 (9.4)	≤ 0.05
Grade 2	5 (11.6)	945 (55.8)	950 (54.7)	
Grade 3	35 (81.4)	590 (34.8)	625 (36)	
Ki67 value^ab^	40.05 ± 22.67	35.92 ± 23.33	–	0.252
Ki67 index category (n = 1738)				
< 15	4 (9.3)	373 (22)	377 (21.7)	0.203
15–24	11 (25.6)	311 (18.3)	322 (18.5)	
25–44	12 (27.9)	406 (24)	418 (24.1)	
> 44	16 (37.2)	605 (35.7)	621 (35.7)	
Size of tumor^ab^	36.77 ± 15.31	36.11 ± 14.84	–	0.825
Tumor stage (n = 660)				
T1	4 (15.4)	83 (13.1)	87 (13.2)	0.695
T2	17 (65.4)	454 (71.6)	471 (71.4)	
T3	5 (19.2)	97 (15.3)	102 (15.5)	
Nodal status (n = 660)				
Positive	12 (46.2)	318 (50.2)	330 (50)	0.689
Negative	14 (53.8)	316 (48.8)	330 (50)	
N stage (n = 660)				
N0	14 (53.8)	320 (50.5)	334 (50.6)	0.431
N1	5 (19.2)	130 (20.5)	135 (20.5)	
N2	1 (3.8)	85 (13.4)	86 (13)	
N3	6 (23.1)	99 (15.6)	105 (15.9)	
Laterality (n = 1738)				
Left	19 (44.2)	865 (51)	884 (50.9)	0.375
Right	24 (55.8)	830 (49)	854 (49.1)	
ER (n = 1738)				
Positive	9 (20.9)	1019 (60.1)	1028 (59.1)	≤ 0.05
Negative	34 (79.1)	676 (39.9)	710 (40.9)	
PR (n = 1738)				
Positive	6 (14)	779 (46)	785 (45.2)	≤ 0.05
Negative	37 (86)	916 (54)	953 (54.8)	
Her2neu (n = 1738)				
Positive	9 (20.9)	742 (43.8)	751 (43.2)	≤ 0.05
Negative	34 (79.1)	953 (56.2)	987 (56.8)	
Lymphocytic infiltration (n = 660)				
Absent	10 (38.5)	364 (57.4)	374 (56.7)	0.132
Moderate	13 (50)	210 (33.1)	223 (33.8)	
Severe	3 (11.5)	60 (9.5)	63 (9.5)	
Insitu component (n = 659)				
Present	11 (42.3)	432 (68.2)	443 (67.2)	≤ 0.05
Absent	15 (57.7)	201 (31.8)	216 (32.8)	
Lymphovascular invasion (n = 660)				
Present	8 (30.8)	157 (24.8)	165 (25)	0.488
Absent	18 (69.2)	477 (75.2)	495 (75)	
Dermal lymphatic invasion (n = 660)				
Present	0 (0)	78 (12.3)	78 (11.8)	0.061
Absent	26 (100)	556 (87.7)	582 (88.2)	
Pagetoid spread (n = 660)				
Present	0 (0)	14 (2.2)	14 (2.1)	1.000
Absent	26 (100)	620 (97.8)	646 (97.9)	
Molecular subtypes(n = 1738)				
Triple negative	29 (67.4)	298 (17.6)	327 (18.8)	≤ 0.05
HER 2	5 (11.6)	363 (21.4)	368 (21.2)	
Luminal A	1 (2.3)	287 (16.9)	288 (16.6)	
Luminal B	8 (18.6)	747 (44.1)	755 (43.4)	

Chi Square applied

^a^Mean ± SD

^b^Independent t-Test

Metaplastic carcinomas were found to be of higher grade as compared to ductal carcinoma NOS (81% of metaplastic carcinoma were grade III compared to 35% ductal carcinoma, NOS). Similarly, metaplastic carcinoma differs with ductal carcinoma NOS in terms of ER/PR positivity. 79 and 86% of metaplastic carcinoma were ER and PR negative, respectively as compared to ductal carcinoma NOS (40 and 54% were ER and PR negative, respectively). No significant difference was noted in respect to T-stage and N-stage of metaplastic carcinoma as compared to ductal carcinoma NOS. Lack of insitu component was noted in more than half of cases of metaplastic carcinoma which differs significantly from ductal carcinoma NOS. Majority of metaplastic carcinomas were triple negative (67.4%) compared to ductal carcinoma NOS, most of which are luminal B (44%).

Table 2 shows the comparison of various clinico-pathologic features of micropapillary carcinoma with ductal carcinoma NOS. No significant association was noted except for ER and PR positivity, where micropapillary cancers were noted to have more frequent positivity of these biomarkers (micropapillary cancers were 86.7 and 73.3% ER and PR positive compared to 60 and 46% in ductal carcinoma NOS).

**Table 2:** Association of clinico-pathologic parameters of micropapillary breast cancer subtype with ductal carcinoma, NOS

	n (%)			P-value
	Micropapillary	Ductal, NOS	Total	
Age (years)^ab^	48.20 ± 14.02	50.54 ± 12.48	–	0.47
Age group (n = 1710)				
≤ 30	2 (13.3)	89 (5.3)	91 (5.3)	0.323
31–50	7 (46.7)	822 (48.5)	829 (48.5)	
51–70	5 (33.3)	692 (40.8)	697 (40.8)	
> 70	1 (6.7)	92 (5.4)	93 (5.4)	
Tumor grade (n = 1710)				
Grade 1	0 (0)	160 (9.4)	160 (9.4)	0.386
Grade 2	11 (73.3)	945 (55.8)	956 (55.9)	
Grade 3	4 (26.7)	590 (34.8)	594 (34.7)	
Ki67 value^ab^	35.80 ± 22.94	35.92 ± 23.33	–	0.984
Ki67 index category (n = 1710)				
< 15	2 (13.3)	373 (22)	375 (21.9)	0.582
15–24	4 (26.7)	311 (18.3)	315 (18.4)	
25–44	5 (33.3)	406 (24)	411 (24)	
> 44	4 (26.7)	605 (35.7)	609 (35.6)	
Size of tumor^ab^	33.11 ± 14.77	36.11 ± 14.84	–	0.547
Tumor stage (n = 643)				
T1	1 (11.1)	83 (13.1)	84 (13.1)	1.000
T2	7 (77.8)	454 (71.6)	461 (71.7)	
T3	1 (11.1)	97 (15.3)	98 (15.2)	
Nodal status (n = 643)				
Positive	6 (66.7)	318 (50.2)	324 (50.4)	0.505
Negative	3 (33.3)	316 (49.8)	319 (49.6)	
N stage (n = 643)				
N0	4 (44.4)	320 (50.5)	324 (50.4)	0.417
N1	2 (22.2)	130 (20.5)	132 (20.5)	
N2	0 (0)	85 (13.4)	85 (13.2)	
N3	3 (33.3)	99 (15.6)	102 (15.9)	
Laterality (n = 1710)				
Left	9 (60)	865 (51)	874 (51.1)	0.489
Right	6 (40)	830 (49)	836 (48.9)	
ER (n = 1710)				
Positive	13 (86.7)	1019 (60.1)	1032 (60.4)	≤ 0.05
Negative	2 (13.3)	676 (39.9)	678 (39.6)	
PR (n = 1710)				
Positive	11 (73.3)	779 (46)	790 (46.2)	≤ 0.05
Negative	4 (26.7)	916 (54)	920 (53.8)	
Her2neu (n = 1710)				
Positive	9 (60)	742 (43.8)	751 (43.9)	0.207
Negative	6 (40)	953 (56.2)	959 (56.1)	
Lymphocytic infiltration (n = 643)				
Absent	3 (33.3)	364 (57.4)	367 (57.1)	0.241
Moderate	5 (55.6)	210 (33.1)	215 (33.4)	
Severe	1 (9.5)	60 (9.5)	61 (9.5)	
Insitu component (n = 642)				
Present	2 (22.2)	432 (68.2)	434 (67.6)	≤ 0.05
Absent	7 (77.8)	201 (31.8)	208 (32.4)	
Lymphovascular invasion (n = 643)				
Present	7 (77.8)	157 (24.8)	164 (25.5)	≤ 0.05
Absent	2 (22.2)	477 (75.2)	479 (74.5)	
Dermal lymphatic invasion (n = 643)				
Present	2 (2.5)	78 (12.3)	80 (12.4)	0.311
Absent	7 (77.8)	556 (87.7)	563 (87.6)	
Pagetoid spread (n = 643)				
Present	0 (0)	14 (2.2)	14 (2.2)	1.000
Absent	9 (100)	620 (97.8)	629 (97.8)	
Molecular subtypes (n = 1710)				
Triple negative	1 (6.7)	298 (17.6)	299 (17.5)	≤ 0.05
HER 2	1 (6.7)	363 (21.4)	364 (21.3)	
Luminal A	0 (0)	287 (16.9)	287 (16.8))	
Luminal B	13 (86.7)	747 (44.1)	760 (44.4)	

Chi Square applied

^a^Mean ± SD

^b^Independent t-Test

Although micropapillary cancers were found to be associated with 66.7% frequency of nodal metastasis, however to significant difference was noted when compared to ductal carcinoma NOS. Furthermore, mean ki67 index of micropapillary carcinoma was 35.8 and 26% were of grade III. Her2neu positivity was noted in 60% of cases. Higher frequency of lymphovascular invasion was noted in micropapillary carcinoma (77.8%) compared to ductal carcinoma NOS (24.8%). 86.7% of micropapillary carcinoma were luminal B compared to 44% of ductal carcinoma, NOS.

### Discussion

In the present study we found a low frequency of metaplastic and micropapillary carcinoma, however these rare types were found to be associated with adverse prognostic parameters. Metaplastic carcinomas were noted to have higher grade and lower expression of hormonal receptors, while micropapillary carcinoma showed higher frequency of lymphovascular invasion. Clinico-pathologic evaluation of tumors is principal to predict the outcome and life expectancy of the patients. On account of the recognition of ductal carcinomas and previously classified as not otherwise specified carcinomas, today the rate of treatment failure has been largely reduced [13]. An antecedent study conducted in the year 2014, revealed that the incidence of tumors demonstrating ductal morphology was as high as 83% in the population [15]. Furthermore, a meaningful number of tumors exhibited metaplastic as well as medullary characteristics [14, 15]. On the grounds of our previous findings; in this study we focused on the clinico-pathologic and histopathologic features of the invasive micropapillary breast carcinoma and the invasive metaplastic carcinoma. Alongside studying these clinico-pathologic features of invasive metaplastic and micropapillary breast carcinoma, we also compared the two subtypes with the invasive ductal carcinoma, not otherwise specified of the breast to expand our knowledge and understanding.

According to Weigelt et al. only 25% of carcinomas diagnosed in laboratory settings are histologically ‘special type’ and further classified into 17 pathological types, while the rest are invasive ductal carcinoma not otherwise specified [16]. In congruence with the previous studies, we observed that ductal carcinoma, not otherwise specified were the most prevalent type of breast cancer in our population, constituting 84% of all breast cancers diagnosed in the laboratory, while only a meagre number of cases of metaplastic (2.2%) and micropapillary (0.76%) carcinomas were notable. The incidence of metaplastic carcinoma was found to be less than 1% by previous studies [17–20], however, in the present study, we observed that the incidence of MBC has increased significantly (2.2% in the present study).

In addition, a marked variation in tumor grade and frequency of ER and PR genes was noticed between the metaplastic and ductal carcinoma not otherwise specified, where metaplastic carcinomas were characterized by high tumor grade as compared to IDC NOS. Since tumor grade is one of the predominant histopathologic feature that predicts the outcome of disease [21–23]. It can be said that metaplastic carcinoma is more aggressive in behavior than IDC NOS. Moreover, the ER and PR positivity was significantly low in our study. A previous study also demonstrated that the percentage of ER, PR and HER-2 positivity was less than 10% in metaplastic carcinoma [18]. Our analysis also showed congruence with these findings as HER-2 positivity was found to be 21% in metaplastic carcinomas and 44% in invasive ductal carcinoma, not otherwise specified.

Furthermore, a significant number of metaplastic carcinoma were found to be triple negative (84%), unlike DC, NOS which were predominantly luminal B type. The findings of our study are consistent with other studies which demonstrated that triple negative characteristic was the predominant molecular subtype in metaplastic carcinoma [18, 25]. Due to triple negative characteristic of metaplastic carcinomas, they are regarded as more aggressive type of cancers which are often less-responsive to endocrine therapy [24].

On the other hand, the incidence of invasive micropapillary carcinoma was reported to be less than 2% (ranging from 0.7 to 3%) of all invasive breast carcinomas [24, 25]. In our study, we found that less than 1% of the cases of breast cancers accounted for invasive micropapillary carcinoma. The clinico-pathologic findings of micropapillary carcinoma in our study revealed a high ER and PR positivity ratio as compared with invasive ductal carcinoma, not otherwise specified. ER positivity was visible in 87% of the cases of IMPC, while only 60% of IDC, NOS showed ER positivity. In another study, ER positivity ranged from 19.4 to 90.6% in invasive micropapillary carcinomas [26]. One of the study also indicated that high PR positivity in invasive micropapillary carcinomas is advantageous in terms of survival and attributable to increased life expectancy in these patients. Therefore, these characteristics suggest good prognosis in patients with MIBC as compared to IDC, NOS since high positivity of ER and PR correlates with good prognosis and tumor outcomes [27, 28] Notwithstanding with these correlations, the IMPC is characterized by poor prognosis and aggressive behavior due to high propensity for nodal metastasis and lymphovascular invasion [27, 28], despite of high PR and ER positivity. The findings of our study reaffirm high incidence of lymphovascular invasion, as noted in 78% of micropapillary carcinomas compared to only 25% ductal carcinoma NOS.

In conclusion, invasive micropapillary carcinoma and invasive metaplastic carcinoma are rare breast cancer subtypes accounting for less than 2 and 1% of the breast cancer burden in our population. Both of the types correlate with adverse prognosis and poor outcomes. Owing to high tumor grade and loss of hormonal receptor expression in metaplastic carcinomas, they are believed to be less responsive to conventional therapies. Similarly, frequent lymphovascular invasion in micropapillary carcinoma correlates with aggressive nature.

We recommend larger-scale, population level studies to find the prevalence of the two rare subtypes of the breast cancer in our population since the results of our study cannot be extrapolated to the whole population as it dealt with a single-facility data. A multi-center study can determine the true prevalence of these rare subtypes of breast cancer in our population and then by knowing the adverse prognostic features of these tumors, therapeutic protocols can be developed for locoregional population.

## Limitations

Lack of clinical follow up to determine recurrence and disease free survival was the major limitation of our study. Moreover, molecular studies like oncotype Dx was not performed on these tumors to determine their molecular adverse prognostic features.

## Abbreviations

DC, NOS: invasive ductal carcinoma, not otherwise specified
MBC: metaplastic breast carcinomas
IMBC: invasive micropapillary breast carcinomas
